# Supplementary material for: Post-pubertal developmental trajectories of laryngeal shape and size in humans
Source: Sci Rep. 2023 May 11;13:7673. doi: 10.1038/s41598-023-34347-w (PMC10175495; doi:10.1038/s41598-023-34347-w)
Supplement: Supplementary file 1 — Supplementary Figure S1. [file 41598_2023_34347_MOESM1_ESM.docx]

**
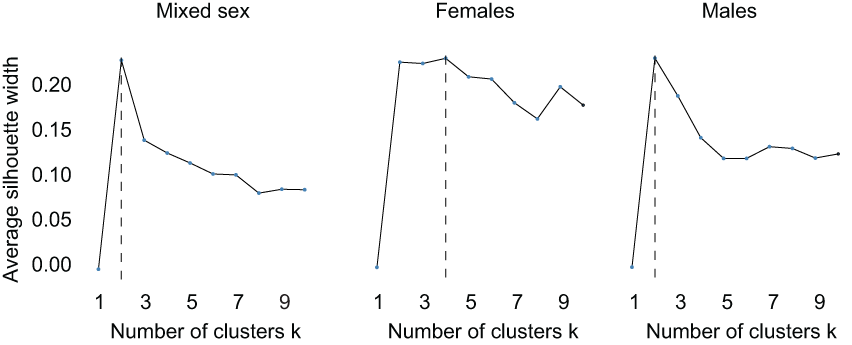
Supplemental Figure S1**: Optimal number of clusters. The silhouette score for males and females combined, recommends 2 clusters. The silhouette analysis (silhouette score) recommends 4 clusters for females and 2 to 3 clusters for males.
